# Plus Ça Change…? How the COVID-19 Crisis May Lead to a Revaluation of the Local

**DOI:** 10.1007/978-3-030-65355-2_13

**Published:** 2021-03-20

**Authors:** Martijn Groenleer, Daniel Bertram

**Affiliations:** 1 Tilburg University, Tilburg, The Netherlands; 2 Tilburg University, Tilburg, The Netherlands; 3 Tilburg University, Tilburg, The Netherlands; 4 Tilburg University, Tilburg, The Netherlands; 5 Department of Public Law and Governance, Tilburg Center for Regional Law and Governance, Tilburg Law School, Tilburg University, The Netherlands; 6 St. Cross College, University of Oxford, Oxford, UK

## Abstract

The spread of the coronavirus around the world once again confronts us with the vulnerability of a globalized economy and society. As with previous global crises, it is not obvious that the COVID-19 crisis is leading to a process of de-globalization. It is much more likely that the crisis will accelerate localization—a process that has been going on for much longer. In fact, localization occurs simultaneously with globalization, and is inextricably bound up with it—in both the “old” and the “new common.” In this sense, the struggle to grapple with the current situation shows us the continuing importance of the local in a global context.

## The normality of global flows - and crises

Who is to blame for the catastrophic economic and social damages (beyond the untold human suffering) that COVID-19 may cause and has already caused? While some have jumped to scapegoating one country (China) or one institution (the WHO), somewhat more sophisticated arguments point in the direction of globalization. Although the economy has been operating in cross-border production chains for centuries as part of a global division of labor, these chains have become increasingly complex and tightly linked in recent decades. As a result, the potential for failure—a chain reaction or even an “infarct”—is considerable, and crises with cross-border effects are inevitable. To some extent, such transboundary crises have become the new common (Boin 2019).

With COVID-19 unfolding its deadly and disruptive force, calls from all across the political spectrum for putting an end to globalization are becoming louder. But will the pandemic really constitute a stumbling block to the inexorable machinery of growing interconnection? We argue that, as with previous global crises such as the financial crisis, it is not obvious that the COVID-19 crisis will lead to a process of de-globalization, however disastrous the consequences of international cross-linking may be for economies and societies. It is much more likely that the new common will see an acceleration of the process of localization, already occurring as part of globalization in the old common.

In the 1990s, sociologist Manuel Castells (1989) spoke in this regard about the creation of a so-called “space of flows.” In the network society, territories and their borders lose much of their importance. As a result of continuous innovations in information and communication technology, locations, or places, are increasingly connected. This phenomenon is mostly economic in nature, but by no means exclusively so. People also organize themselves across borders via the Internet and social media in global social movements: for entertainment, for political purposes, for spiritual themes, and indeed for all kinds of protests. Digital connectivity, therefore, seems to have largely replaced geographical proximity.

## The Enduring Importance of Place

However, it turns out time and time again: the world is not “placeless” suddenly. Even in a globalized society—in spatial terms—concrete places are still linked together. Those loci are the regions, cities, and neighborhoods where we lead our daily lives, derive our identity from, and build communities around. From its very origin, the ravaging COVID-19 crisis illustrates both the importance of the local, and how and where we live forms part of a larger, global whole. After all, the virus was likely first transmitted from animal to human in Wuhan, one of China’s sprawling metropoles and an important national and international hub, from where it quickly conquered all corners of the world. Ever since, cities and urban areas appear at the epicenter of the crisis (Keil et al. 2020).

The result is localization—not so much as the antagonist to globalization, but as part of it. This holds true for both economic and social processes. The crisis has not only propelled the further rise of global players like Amazon and Google but it has also led many to appreciate the availability and indispensability of local goods and services in an unprecedented manner (Vijn 2020). Rarely have the bakery around the corner, the florist next door, and the various local mom-and-pop businesses received this much love and attention. A similar pattern is discernible for social processes. In spite of students and colleagues now being dispersed around the world, their social connections are soaring over distance—thanks to Zoom, Teams, and Hangouts & Co. Such global connectivity has not come at the cost of local solidarity: there is an overwhelming number of genuinely heartwarming news stories of people stepping in for each other (see, e.g., Stewart 2020).

These dynamics have also impacted the sphere of governance. Even though we seem to be witnessing a return of the territorial state during the acute crisis phase, cities, and regions are only gaining weight as scale levels. Behind the scenes, powers have been shifted downwards, e.g., to the 25 mayors of the Dutch security regions (Marijnissen 2020). Beyond implementing and enforcing national measures, cities, and regions shape distinctive responses to globalized issues. The wicked problems and grand challenges of our times may primarily operate at an international level, but the consequences are felt locally, and differently. In the case of COVID-19, starting with differences in death tolls as a result of the virus but going all the way to differences in bankruptcies, unemployment rates, and social conditions. The tragic examples of Wuhan, Milan, New York, Sao Paulo, and, indeed, the Dutch province of Brabant and the German Land of Bavaria have made it clear that not only certain sectors or certain groups but also specific places are being affected unequally.

## The Need for a Differentiated Response

Hence, differentiated policy measures and regulatory responses become necessary to limit contextually specific adverse effects. A one-size-fits-all approach neglects the unique geographic trajectory of COVID-19. It may well result in a one-size-fits-none situation where central measures are insufficient to limit the spread of the virus in some places while, in other places, impose an excessive, unnecessary burden on the local population. This explains the significant variation found globally, within the countries of the European Union, and even within a small country such as the Netherlands in tackling COVID-19. In the Dutch case, the initial response occurred regionally, in the province of Brabant, where the virus made its first nationwide appearance. After increasingly centralized measures were taken by the national authorities during the most severe phases in late March and April 2020, the approach shifted to regional differentiation and local customization. Throughout the summer, Amsterdam had stricter regulations in place than other parts of the country, citing the high population density and the inflow of foreign tourists as aggravating risk factors (Muller 2020).

The Netherlands is hardly an isolated example in this regard. In Germany, the United States, Brazil, and even in France, to name just a few, the regional and local levels have exercised significant influence in the handling of the COVID-19 crisis—on occasion in direct opposition to national positions (Der Spiegel 2020). Sometimes, this depends on institutional factors, such as differing degrees of federalism in Germany, the United States, and Brazil. In addition, local and regional bearing seems to vary in time, just like in the Netherlands: from local measures at the start to centralized restrictions as the crisis develops, and back to local responses when case numbers have somewhat declined and outbreaks can be locally monitored and controlled. But even across different political systems and across different stages in the infamous curve of infections, subnational voices are loud and their actions decisive.

The same dynamic extends beyond immediate policies to measures targeted at the virus’s more mediate impacts. Whereas national governments are busy saving multinational businesses from bankruptcy, this is the time for local and regional authorities to shine as policy entrepreneurs. Urban governments in Berlin, Milan, Brussels, and elsewhere are now designating new bike lanes in reaction to decreased car traffic (Curry 2020). The Ecuadorian capital of Quito enacted new rules for the city’s critical food markets to safeguard urban food security while limiting the risk of infection for the many consumers and workers (Rodríguez 2020). The city of San Francisco, like many others, is providing emergency shelters to the homeless in an effort to improve sanitary conditions and enable effective social distancing (Ho 2020).

## The Local as Part of the Global: Glocality

It was another sociologist, Roland Robertson (1995), who was one of the first to use the term “glocalization” in the mid-1990s to describe the simultaneous occurrence of processes of globalization and localization. Many developments in our economy are aimed at standardization and upscaling. At the same time, there is an increasing need to develop customized solutions and to adapt to local conditions, also socially and politically. Indeed, the term glocalization has its origins in the business world and the adaptation of standardized products and services to local needs. Think of the “McKroket,” a special food product launched by the globally operating fast-food chain McDonalds to serve the Dutch affinity for the deep-fried snack.

The counterintuitive beauty of this dynamic is that the global is not so much the opposite of the local. On the contrary, what is often called the local is essentially part of the global. Globalization can thus be seen as the shrinking of the world by connecting places. In fact, we are seeing that this is accelerated. A notable example is the initiative of regional and local leaders to share knowledge and facilitate mutual policy learning during the pandemic, across national, cultural, and language borders, as in UNESCO’s (2020) Global Network of Learning Cities, the Harvard Bloomberg City Leadership Initiative (Harvard Kennedy School 2020), or the City of Amsterdam’s (2020) “International Monitor”. Incidentally, it is by no means fixed or predetermined what constitutes a place: the process of localization also includes the “(re)invention of place.” And this is what we currently seem to be witnessing, by both citizens and those responsible for designing and implementing policy measures and regulatory responses.

Hence, glocalization links two apparently contradictory processes and emphasizes this form of complexity in contemporary society. The term can also help to interpret current processes related to the COVID-19 crisis, including in governance. The tasks faced are generic and transboundary in various respects, while locally specific and context-dependent in others. Essentially, the various local and regional responses, similar but different, are the socio-political McKrokets of a world in crisis.

## In Sum: … Plus C’est la Même Chose?

The spread of the coronavirus around the world once again confronts us with the vulnerability of a globalized economy and society. Even though globalization may have contributed to the spread of the virus, it is far from obvious that this will lead to a process of de-globalization in the longer term. At the moment, there may be a significant dip in global economic interactions, but the space of flows is likely to keep growing. In line with this, COVID-19 seems to enhance the simultaneous localization process that has been ongoing for much longer as part of a concerted attack on the authority of the territorial state. The space of places has returned, or rather it has never gone away. If anything, the struggle to grapple with the current situation could contribute to a revaluation of the local in a global context. In this sense, rather than a disruption, the new common may actually turn out to be no more (and no less) than a slightly altered, yet considerably accelerated version of the old common.
